# 129 Are There Differences in Scar Assessments as a Function of Fitzpatrick Skin Type

**DOI:** 10.1093/jbcr/irae036.128

**Published:** 2024-04-17

**Authors:** Bonnie C Carney, Davon T Lee, Taryn E Travis, Rebekah R Allely, Shawn Tejiram, Jeffrey W Shupp

**Affiliations:** MedStar Health Research Institute, Washington, District of Columbia; Firefighters Burn and Surgical Labratory of Washington Hospital Medical Center and Georgetown University., Waldorf, Maryland; MedStar Washington Hospital Center, Georgetown University School of Medicine, Washington, DC; Medstar Washington Hospital Center, Washington, District of Columbia; Georgetown University School of Medicine, Washington, DC; MedStar Health |Georgetown University School of Medicine, Washington, DC; MedStar Health Research Institute, Washington, District of Columbia; Firefighters Burn and Surgical Labratory of Washington Hospital Medical Center and Georgetown University., Waldorf, Maryland; MedStar Washington Hospital Center, Georgetown University School of Medicine, Washington, DC; Medstar Washington Hospital Center, Washington, District of Columbia; Georgetown University School of Medicine, Washington, DC; MedStar Health |Georgetown University School of Medicine, Washington, DC; MedStar Health Research Institute, Washington, District of Columbia; Firefighters Burn and Surgical Labratory of Washington Hospital Medical Center and Georgetown University., Waldorf, Maryland; MedStar Washington Hospital Center, Georgetown University School of Medicine, Washington, DC; Medstar Washington Hospital Center, Washington, District of Columbia; Georgetown University School of Medicine, Washington, DC; MedStar Health |Georgetown University School of Medicine, Washington, DC; MedStar Health Research Institute, Washington, District of Columbia; Firefighters Burn and Surgical Labratory of Washington Hospital Medical Center and Georgetown University., Waldorf, Maryland; MedStar Washington Hospital Center, Georgetown University School of Medicine, Washington, DC; Medstar Washington Hospital Center, Washington, District of Columbia; Georgetown University School of Medicine, Washington, DC; MedStar Health |Georgetown University School of Medicine, Washington, DC; MedStar Health Research Institute, Washington, District of Columbia; Firefighters Burn and Surgical Labratory of Washington Hospital Medical Center and Georgetown University., Waldorf, Maryland; MedStar Washington Hospital Center, Georgetown University School of Medicine, Washington, DC; Medstar Washington Hospital Center, Washington, District of Columbia; Georgetown University School of Medicine, Washington, DC; MedStar Health |Georgetown University School of Medicine, Washington, DC; MedStar Health Research Institute, Washington, District of Columbia; Firefighters Burn and Surgical Labratory of Washington Hospital Medical Center and Georgetown University., Waldorf, Maryland; MedStar Washington Hospital Center, Georgetown University School of Medicine, Washington, DC; Medstar Washington Hospital Center, Washington, District of Columbia; Georgetown University School of Medicine, Washington, DC; MedStar Health |Georgetown University School of Medicine, Washington, DC

## Abstract

**Introduction:**

Skin type, as organized within the Fitzpatrick scale, may be an important variable in scar severity. Fitzpatrick skin type II (FST-II) and Fitzpatrick skin type V (FST-V) were chosen, to compare scar outcomes of lighter and darker skin patients with burn injuries. It was hypothesized that patients with FST-V skin have worse scar outcomes than patients with FST-II skin.

**Methods:**

Demographic variables were obtained from the EMR including patient age, age of scar at time of assessment, gender, etiology of burn injury, and location of injury for this retrospective chart review. A variety of scar assessment scales and non-invasive measurements of scar qualities were administered during visits with the rehabilitation team around 4 months post-burn. These included The Patient and Observer Scar Assessment Scale (POSAS) which was broken up into: POSAS-Observer (O) avg, POSAS-Patient (P) Itch, POSAS-P Pain, and POSAS-P avg. This evaluation also included the Vancouver Scar Scales (VSS), and pliability, erythema, and melanin objective measurements.

**Results:**

Thirty patients were included in the analysis, 15 in each group. In FST-II, 53% of subjects were female, 47% were male. In FST-V, 33% of patients were male, while 67% were female (p>0.05). There are no significant differences in categorical patient age ( 42.2±4.6 vs 45.6±4). Approximately 67% of FST-II patients had flame burns, 33% scald injuries. Similarly 62% of FST-V subjects had flame burns, and 38% had scald injuries. The location of injuries was variable in both groups, but not different among groups. Scars were evaluated at 3.7±0.3 months with a range from 1-8 months post-burn and this evaluation time was not different between groups. For observer driven scar assessment (POSAS-O avg and VSS), there were no differences in scores between the groups (6.2±0.4 vs. 6.1±0.4 and 6.1±0.4 vs. 5.8±0.5). POSAS-P Itch scores and Melanin levels were higher in FST-V (7.3±0.6 vs. 5.3±0.7, p< 0.05, 896.4±9.5 vs. 727.2±9.8, p< 0.0001). Erythema was higher in FST-II (463.7±6.4 vs. 398.7±11.4, p< 0.0001). There was no statistical difference in POSAS-P Pain or pliability scores between FST-II and FST-V groups.

**Conclusions:**

These findings suggest that patients rate their scars as being equally morbid regardless of skin type. There may be a correlation between higher melanin levels and pruritis in burn scars. Although scars in darker patients is thought to result in worse outcomes, the data shows that observers rate them equally. These findings bring into question the validity of current scar assessment scales and whether they adequately measure scars within different FST groups.

**Applicability of Research to Practice:**

Performance of current scar scales in different skin types could affect treatment decisions. New metrics may need to be added to current scar assessment tools.

